# The damage and tolerance mechanisms of *Phaffia rhodozyma* mutant strain MK19 grown at 28 °C

**DOI:** 10.1186/s12934-020-01479-x

**Published:** 2021-01-07

**Authors:** Li-Li Miao, Shuang Chi, Ting-Ting Hou, Zhi-Pei Liu, Ying Li

**Affiliations:** 1 State Key Laboratory of Microbial Resources, Institute of Microbiology, Chinese Academy of Sciences, Beijing, 100101 People’s Republic of China; 2 State Key Laboratories for Agro-biotechnology and College of Biological Sciences, China Agricultural University, Beijing, 100193 People’s Republic of China

**Keywords:** *Phaffia rhodozyma*, Moderate-temperature strain, Astaxanthin, Fatty acid, Mevalonate pathway

## Abstract

**Background:**

*Phaffia rhodozyma* has many desirable properties for astaxanthin production, including rapid heterotrophic metabolism and high cell densities in fermenter culture. The low optimal temperature range (17–21 °C) for cell growth and astaxanthin synthesis in this species presents an obstacle to efficient industrial-scale astaxanthin production. The inhibition mechanism of cell growth at > 21 °C in *P. rhodozyma* have not been investigated.

**Results:**

MK19, a mutant *P. rhodozyma* strain grows well at moderate temperatures, its cell growth was also inhibited at 28 °C, but such inhibition was mitigated, and low biomass 6 g/L was obtained after 100 h culture. Transcriptome analysis indicated that low biomass at 28 °C resulted from strong suppression of DNA and RNA synthesis in MK19. Growth inhibition at 28 °C was due to cell membrane damage with a characteristic of low mRNA content of fatty acid (f.a.) pathway transcripts (*acc*, *fas*1, *fas*2), and consequent low f.a. content. Thinning of cell wall and low mannose content (leading to loss of cell wall integrity) also contributed to reduced cell growth at 28 °C in MK19. Levels of astaxanthin and ergosterol, two end-products of isoprenoid biosynthesis (a shunt pathway of f.a. biosynthesis), reached 2000 µg/g and 7500 µg/g respectively; ~2-fold higher than levels at 21 or 25 °C. Abundance of ergosterol, an important cell membrane component, compensated for lack of f.a., making possible the biomass production of 6 g/L for MK19 at 28 °C.

**Conclusions:**

Inhibition of growth of *P. rhodozyma* at 28 °C results from blocking of DNA, RNA, f.a., and cell wall biosynthesis. In MK19, abundant ergosterol made possible biomass production 6 g/L at 28 °C. Significant accumulation of astaxanthin and ergosterol indicated an active MVA pathway in MK19 at 28 °C. Strengthening of the MVA pathway can be a feasible metabolic engineering approach for enhancement of astaxanthin synthesis in *P. rhodozyma*. The present findings provide useful mechanistic insights regarding adaptation of *P. rhodozyma* to 28 °C, and improved understanding of feasible metabolic engineering techniques for industrial scale astaxanthin production by this economically important yeast species.

## Background

Reactive oxygen species (ROS), which are generated in both biochemical and photochemical systems [1], cause cell damage by oxidizing biomolecules such as DNA, proteins, and lipids. Astaxanthin, an orange-red carotenoid pigment, acts as a protective agent against the effects of oxidative damage to cells in vivo [2, 3]. The antioxidant activity of astaxanthin is 100- to 500-fold greater than that of vitamin E [4–8]. Astaxanthin from dietary sources is responsible for the orange-red coloration of salmon, lobster, and other seafood species, which cannot synthesize the compound de novo. Astaxanthin, particularly that from natural sources, is utilized extensively in the food, aquaculture, cosmetic, and pharmaceutical industries because of its distinctive coloration and antioxidant properties [1, 9–12].

*Phaffia rhodozyma* (sexual form: *Xanthophyllomyces dendrorhous*), a carotenoid-synthesizing yeast having astaxanthin as the main pigment, is the most promising and economical natural source of astaxanthin and has great industrial potential for astaxanthin fermentation [13, 14]. *P. rhodozyma* was initially isolated from exudates of trees in isolated locations in Japan and the West Coast of North America. Because its native environment is fairly cold, *P. rhodozyma* is a psychrophilic (low temperature preferring) species. Both cell growth and astaxanthin biosynthesis in *P. rhodozyma* are inhibited by high temperatures [15]. The optimal temperature range for these processes is between 17 and 21 °C, and this fact presents an obstacle to industrial production of astaxanthin [15].

Astaxanthin and ergosterol are isoprenoid compounds sharing the conserved mevalonate (MVA) pathway in *P. rhodozyma* [16]. All isoprenoid compounds are based on the C5 isoprene unit. In the initial step of the MVA pathway, two molecules of acetyl-CoA (which is also a substrate of the fatty acid synthetic pathway) undergo condensation to yield acetoacetyl-CoA, which is then converted to MVA, catalyzed by the *hmgs* and *hmgr* gene products. MVA is then transferred to farnesyl pyrophosphate (FPP) through a series of condensation steps catalyzed by *mvk*, *mpd*, *idi*, and *fps*-encoded enzymes. This process is collectively termed the MVA pathway because of the importance of the metabolite MVA. FPP undergoes reduction catalyzed by two specific enzymes, encoded by *sqs* [17] and *crt*E, giving rise respectively to ergosterol and astaxanthin.

Formation of phytoene from geranylgeranyl diphosphate (GGPP) is catalyzed by phytoene-β-carotene synthase (encoded by *pbs*), a bifunctional enzyme that also displays lycopene cyclase function in *P. rhodozyma* [18, 19]. Lycopene is synthesized from phytoene through four subsequent dehydration steps catalyzed by the *crt*I gene product [20]. β-carotene is generated by introducing ring structures at both ends of lycopene, also catalyzed by phytoene-β-carotene synthase [19]. Finally, β-carotene is hydroxylated and oxidized to astaxanthin by astaxanthin synthase, which is encoded by the single gene *ast* [21, 22].

Numerous studies during the past decade, by our lab and others, have addressed the molecular regulatory mechanisms of cell growth and astaxanthin synthesis in *P. rhodozyma* [23–28]. However, these mechanisms remain poorly understood; in particular, the regulatory effects of temperature have not been investigated.

In our previous studies, an astaxanthin-overproducing mutant strain of *P. rhodozyma* termed MK19 that grows well at moderate temperature (25 °C) was generated by 1-methyl-3-nitro-1-nitrosoguanidine (NTG) and Co60 mutagenesis, and its properties were evaluated [23–26]. In the present study, we examined the alterations in cell growth and synthesis of isoprenoids, cell wall, fatty acids, etc. that occur in MK19 during 28 °C stress, a temperature at which wild-type (WT) *P. rhodozyma* strain JCM9042 is unable to grow. The molecular effect of high temperature (28 °C) on regulation of cell growth and astaxanthin synthesis was investigated through comparison of transcriptional profiling response to 21 and 28 °C conditions in WT and MK19.

The obtained transcriptome and metabolic data provide new insights into the genetic and physiological traits and tolerance mechanisms of *P. rhodozyma*, and also reveal potential bioprocesses for optimization of industrial-scale cell growth and astaxanthin synthesis.

## Results

Mutant strain MK19 grew at 28 °C, Astaxanthin content was enhanced significantly at 28 °C in MK19.

**Fig. 1:**
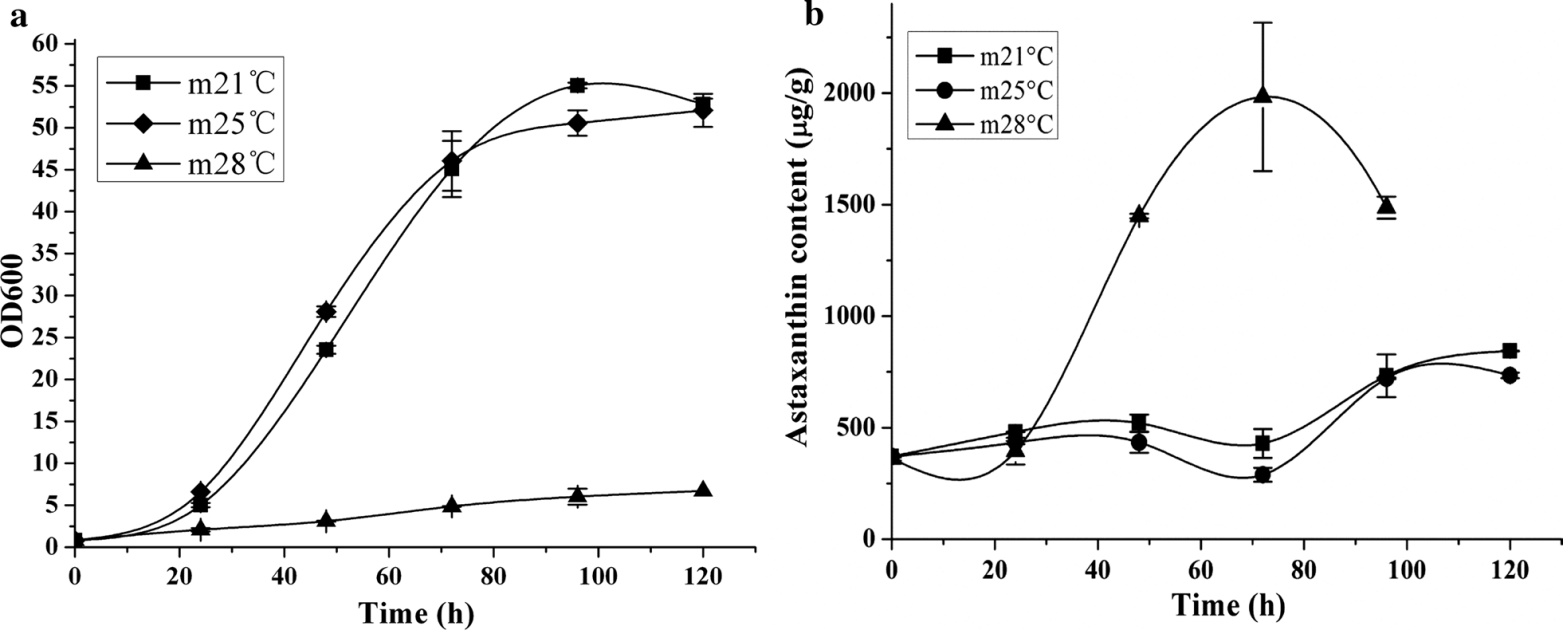
Cell growth (**a**) and astaxanthin content (**b**) of *P. rhodozyma* strain MK19 at three temperatures as indicated

In our 2010 study, *P. rhodozyma* WT strain JCM9042 grew optimally in the temperature range 17–21 °C, more slowly at 25 °C [23], and did not grow at 28 °C. Moderate-temperature mutant MK19 grew as well at 25 °C as it did at 21 °C [23]. In the present study, growth of MK19 were inhibited but not disrupted at temperatures > 25 °C, and biomass production reached 6 g/L for 100 h culture at 28 °C (Fig. 1a). The temperature resulting in complete suppression of MK19 growth was higher than that for WT.

For WT, astaxanthin synthesis was reduced and cell coloration nearly eliminated at temperatures > 25 °C [23]. For MK19, in contrast, astaxanthin content at 28 °C was > 2000 µg/g, which was ~ 2-fold higher than content at 21 or 25 °C (Fig. 1b). Astaxanthin volume yield at 28 °C was low because of limitation of biomass. For MK19, astaxanthin synthesis tolerated a temperature of 28 °C as well as it did 25 °C. These findings suggest that cell growth and astaxanthin synthesis in *P. rhodozyma* are controlled by temperature through independent mechanisms.

### Transcriptional profiling of MK19 under 28 °C stress

We used RNA-Seq to investigate genomic transcription changes in MK19 during response to 28 °C and the regulatory network activated by 28 °C stress. Heat shock response (HSR) functions as a molecular chaperone to protect thermally damaged proteins from aggregation, to unfold aggregated proteins, and to refold damaged proteins or target them for efficient degradation. For validation of RNA-Seq data, we performed RT-PCR analysis of 30 genes involved in heat shock-related response, MVA synthetic pathway, and astaxanthin synthetic pathway. For each of these genes, expression was strongly correlated (correlation coefficient 0.86) with RNA-Seq data. Transcripts of twelve heat shock-related genes coding heat shock proteins (HSPs) such as HSP70, HSP30, HSP60, HSP90, HSP78, and HSP104 were notably upregulated under 28 °C treatment (Table 1). These findings suggest that HSR, which induces a battery of cytoprotective genes that encode HSPs, is an adaptive mechanism in MK19.

**Table 1:** Fold expression changes of HSP, f.a., etc. related genes at 28 vs. 21 °C, based on RT-PCR and RNA-Seq analyses

	Gene	Function	RNA-Seq: fold-change 28/21 °C	RT-PCR: fold-change 28/21 °C
1	comp12727_c0	HSP	1.75	1.93
2	comp13502_c0	HSP homolog pss1	16.16	3.87
3	comp12521_c0	DnaJ family	1.25	-1.29
4	comp11731_c2	HSP	2.25	8.65
5	comp11698_c0	Stress-induced protein STI1	2.63	2.33
6	comp12783_c0	HSP104	2.58	4.35
7	comp12308_c0	HSP90	12.06	3.60
8	comp11869_c0	HSP60	2.77	2.71
9	comp12396_c0	HSP78	2.97	4.50
10	comp10325_c0	HSP SSB	2.45	2.23
11	comp11696_c0	hsp70-like protein	3.74	3.65
12	comp12989_c0	HSP70	5.66	N.D.
13	comp13599_c0	FAS2	0.29	N.D.
14	comp13900_c0	FAS1	0.40	N.D.
15	comp13834_c0	ACC1, acetyl-CoA carboxylase	0.25	N.D.
16	comp11956_c0	f.a.-2 hydroxylase	0.48	N.D.
17	comp12194_c0	f.a. desaturase	0.32	N.D.
18	comp10129_c0	WSC domain-containing protein	0.03	N.D.
19	comp10153_c0	WSC	1.80	N.D.
20	comp10598_c1	WSC domain-containing protein	0.18	N.D.
21	comp10909_c0	Related to WSC2 glucoamylase III	0.77	N.D.
22	comp10944_c0	WSC domain-containing protein	0.03	N.D.
23	comp11101_c1	WSC	1.52	N.D.
24	comp11381_c0	WSC domain-containing protein	0.02	N.D.
25	comp11733_c0	WSC	0.30	N.D.
26	comp12382_c1	WSC-domain-containing protein	0.04	N.D.
27	comp12404_c0	WSC	0.15	N.D.
28	comp12789_c0	WSC	0.82	N.D.
29	comp12838_c0	WSC	0.11	N.D.
30	comp11864_c1	Alpha-1,3-mannosyltransferase CMT1	2.82	N.D.
31	comp12286_c0	Glycosyltransferase family 22 protein	3.88	N.D.
32	comp12082_c1	Alpha-1,6-mannosyltransferase	0.53	N.D.
33	comp11585_c0	Glycosyltransferase family 22 protein	1.95	N.D.
34	comp13450_c0	1,3-beta-glucanosyltransferase	0.45	N.D.
35	comp11848_c1	Endo-1,3(4)-beta-glucanase	0.27	N.D.
36	comp11848_c2	Endo-1,3(4)-beta-glucanase	0.31	N.D.
37	comp11853_c0	Endo-1,3(4)-beta-glucanase	2.89	N.D.
38	comp12961_c0	Glucan endo-1,3-alpha-glucosidase agn1	0.33	N.D.
39	comp10598_c0	Glycoside hydrolase family 71 protein	0.14	N.D.
40	comp13745_c1	Chitin deacetylase	4.50	N.D.
41	comp14086_c0	Glycosyltransferase family 2 protein	2.41	N.D.
42	comp12056_c0	Chitin synthase 1	1.75	N.D.
43	comp12623_c0	Glycosyltransferase family 2 protein	1.99	N.D.
44	comp13627_c0	Glycosyltransferase family 2 protein	2.2	N.D.
45	comp13980_c0	Chitin synthase 6	2.2	N.D.
46	comp11755_c0	HMG-CoA synthase A	0.80	1.39
47	comp13410_c0	HMG-CoA reductase	1.03	1.08
48	comp13164_c0	FPP synthase	1.12	1.50
49	comp10042_c0	IPP isomerase	0.87	2.78

*N.D.* not detected

No. 1–12: HSP genes. No. 13–17: f.a. biosynthetic pathway genes. No. 18–29: WSC genes. No. 30–33: mannan biosynthetic pathway genes. No. 34–39: glucan biosynthetic pathway genes. No. 40–45: chitin biosynthetic pathway genes.No. 46–49: MVA pathway genes

**Fig. 2:**
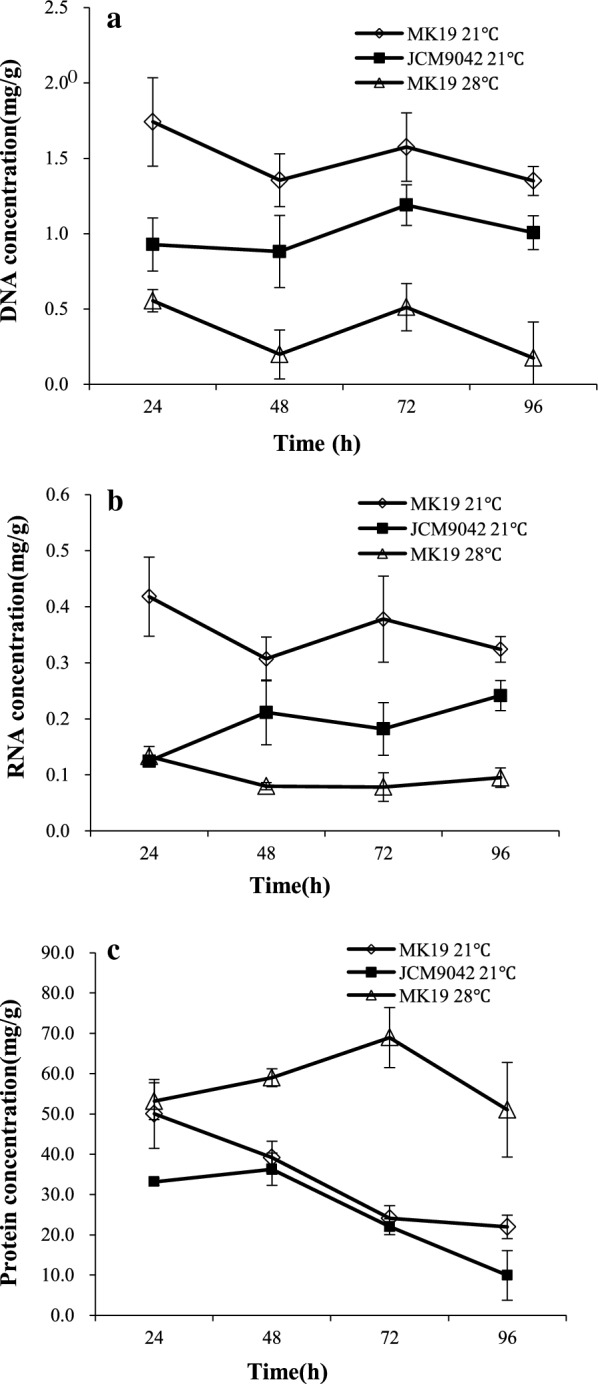
Concentrations of DNA (**a**), RNA (**b**), and protein (**c**) in MK19 grown at 21 and 28 °C, and in strain JCM9042 (WT) grown at 21 °C

Initial functional classification of these differentially expressed genes, using Gene Ontology (GO) and KEGG enrichment, showed that the “purine metabolism” and “pyrimidine metabolism” subsets contained the highest number of genes differentially expressed during MK19 exposure to 28 °C stress. In the “purine” subset, 24 out of 25 differentially expressed genes showed significant upregulation. In the “pyrimidine” subset, 16 out of 20 differentially expressed genes were upregulated under 28 °C treatment. Several subsets of genes involved in rRNA and amino acid metabolic processing were also upregulated at 28 °C. Protein contents were > twohold higher under 28 °C treatment than under 21 °C treatment throughout the culture period (Fig. 2c), while the RNA synthesis was suppressed at 28 °C (Fig. 2b).

The genes downregulated under 28 °C treatment belonged mostly to the “base excision repair” and “fatty acid synthesis” subsets. Seven out of 8 differentially expressed genes in the “base excision repair” subset had extremely low mRNA content at 28 °C, and DNA content was > threefold lower at 28 °C than at 21 °C (Fig. 2a). These findings indicate that the low biomass of MK19 at 28 °C was due to strong suppression of DNA and RNA metabolism.

### MK19 cell membrane was damaged at 28 °C

**Fig. 3:**
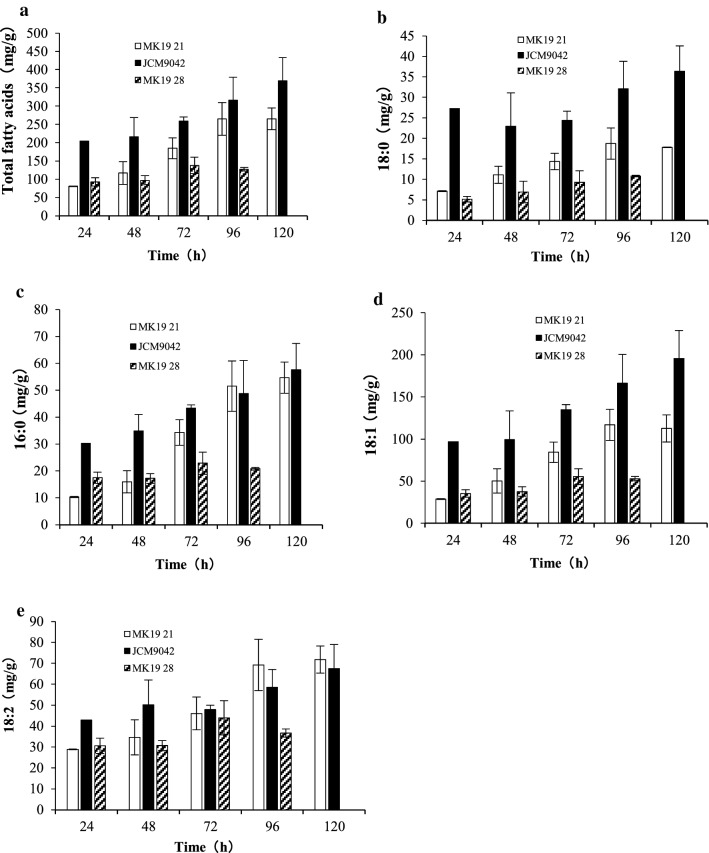
Concentrations of total f.a. (**a**), and f.a. species 18:0 (**b**), 16:0 (**c**), 18:1 (**d**), and 18:2 (**e**) in MK19 grown at 21 and 28 °C, and in JCM9042 at 21 °C

Biological membranes function as permeable or semi-permeable barriers and play key roles in a variety of physiological processes. Maintenance of proper membrane function depends on a precise balance of various lipid species. The biosynthetic pathway of fatty acids (f.a.), essential component of cell membranes, competes with the astaxanthin biosynthetic pathway for the precursor acetyl coenzyme A. Synthesis of f.a., particularly 18:0, 16:0, and 18:1 f.a., was significantly lower at 28 °C than 21 °C (Fig. 3). These f.a. species declined steadily throughout the culture period, particularly after 48 h. Total f.a. content at 28 °C was ~ 50% of that detected at 21 °C treatment (Fig. 3a). 18:1 is the most abundant f.a. species in *P. rhodozyma* (Fig. 3d), and the inhibitory effect of temperature on f.a. synthesis is based mainly on control of 18:1 synthesis. These findings indicate that reduced biomass of *P. rhodozyma* mutant strain MK19 is due in part to deficiency of f.a., mainly 18:1.

**Fig. 4:**
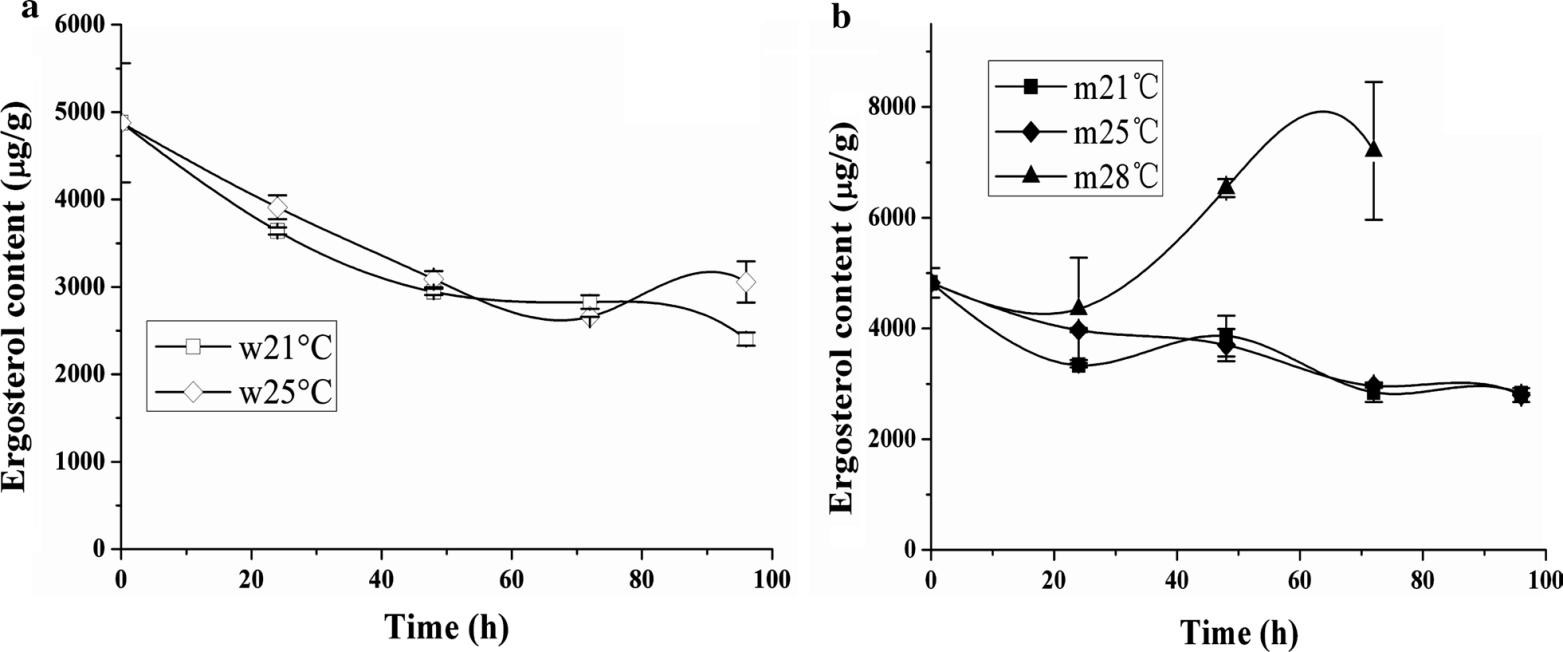
Ergosterol contents of JCM9042 (**a**) and MK19 (**b**) grown at three temperatures

Ergosterol synthesis shares with carotenoid synthesis the initial biosynthetic steps. Changes in sterol composition are associated with enhanced thermotolerance in yeast [29]. WT and MK19 did not show notable differences in ergosterol content at 21 vs. 25 °C. However, astaxanthin content in WT was lower at 25 than at 21 °C [23]. Regulation of terpenoids and sterols by temperature is evidently based on different mechanisms; only terpenoid synthesis was inhibited specifically by 25 °C in WT *P. rhodozyma*. In contrast, ergosterol content was significantly different at 28 °C in comparison with 21 °C; that of MK19 was nearly twofold higher at 28 °C than at 25 or 21 °C (Fig. 4b). Astaxanthin synthesis was also higher at 28 °C in MK19 (Fig. 1b). Ergosterol is an important structural enhancement (strengthening) component of cell membranes. Promotion of ergosterol synthesis in MK19 compensated the reducing of fatty acids and mitigates inhibition of cell growth and helps modulate adaptive response to 28 °C stress. High temperature apparently fosters sterol and terpenoid metabolic fluxes simultaneously in MK19.

According to RNA-Seq analysis, genes involved in f.a. synthetic pathway had very low mRNA content at 28 °C. mRNA content at 28 °C for *acc*1(comp13834_c0), the first key regulatory gene in f.a. synthetic pathway [26], was ~ 25% relative to 21 °C. Low transcription of *acc*1 in MK19 at 28 °C accounts for the low f.a. content and to some degree the low biomass at this temperature. Besides *acc*1, levels of *fas*1 (comp13900_c0), *fas*2 (comp13599_c0), f.a.-2 hydroxylase (comp11956_c0), and f.a. desaturase (comp12194_c0) were reduced at 28 °C (Table 1, No. 13–16). Metabolic and mRNA data, taken together, indicate that f.a. synthetic pathway was hindered by 28 °C stress, and that growth of *P. rhodozyma* at 28 °C requires an adequate amount of f.a. Therefore, modification of *acc*, *fas*1, and *fas*2 expression in future studies could potentially enhance cell growth at 28 °C. *sqs* is the first key regulatory gene in ergosterol synthetic pathway [26]. *sqs* and other genes in this pathway showed no notable change in mRNA content. In contrast to mRNA content, ergosterol content of MK19 was 2-fold higher at 28 °C than at 21 or 25 °C. Increased content of ergosterol may compensate in part for loss of f.a., and promote survival of MK19 at 28 °C.

Another relevant factor is the competition among carotenoids, ergosterol, f.a., and other macromolecules for acetyl-CoA and FPP. When f.a. synthesis was suppressed by high temperature in MK19, a large amount of the intermediate acetyl-CoA was likely accumulated and transferred to isoprenoid biosynthetic pathway through upstream MVA pathway, with the result that astaxanthin and ergosterol content were 2-fold higher at 28 °C than at lower temperatures. This observation is consistent with the conclusion from our 2011 study that strengthening of MVA pathway in MK19 is a promising metabolic engineering approach for enhancement of astaxanthin production [24]. In the present study, carotenoid content was inversely correlated with f.a. biosynthesis.

#### Suppression of cell wall metabolites contributes to reduced cell growth at 28 °C

The fungal cell wall plays an essential role in maintenance of cell shape, integrity, and function. It contacts and interacts with the extracellular environment, and can trigger various physiological processes to adapt to changing circumstances. WSC1, a stress circumstance sensory protein located in cell membrane, is used as a probe for cell wall functioning in fungi. Increasing evidence indicates that defects in *wsc1* and other *wsc* family genes in yeast contribute to increased sensitivity to temperature or other stress factors, and may lead to cell lysis [30, 31]. In the present study, none of the *wsc* genes showed mRNA increase. Under 28 °C treatment of MK19, 8 out of 12 Wsc domain-containing proteins showed extremely low mRNA, one (comp11733_c0) showed 3.5-fold downregulation, and others showed 8- to 32-fold downregulation (Table 1, No. 18–29). These findings suggest that temperature sensitivity in MK19 is related to low mRNA level of *wsc* genes, and that high temperature suppresses cell growth through its effect on cell wall synthesis.

**Fig. 5:**
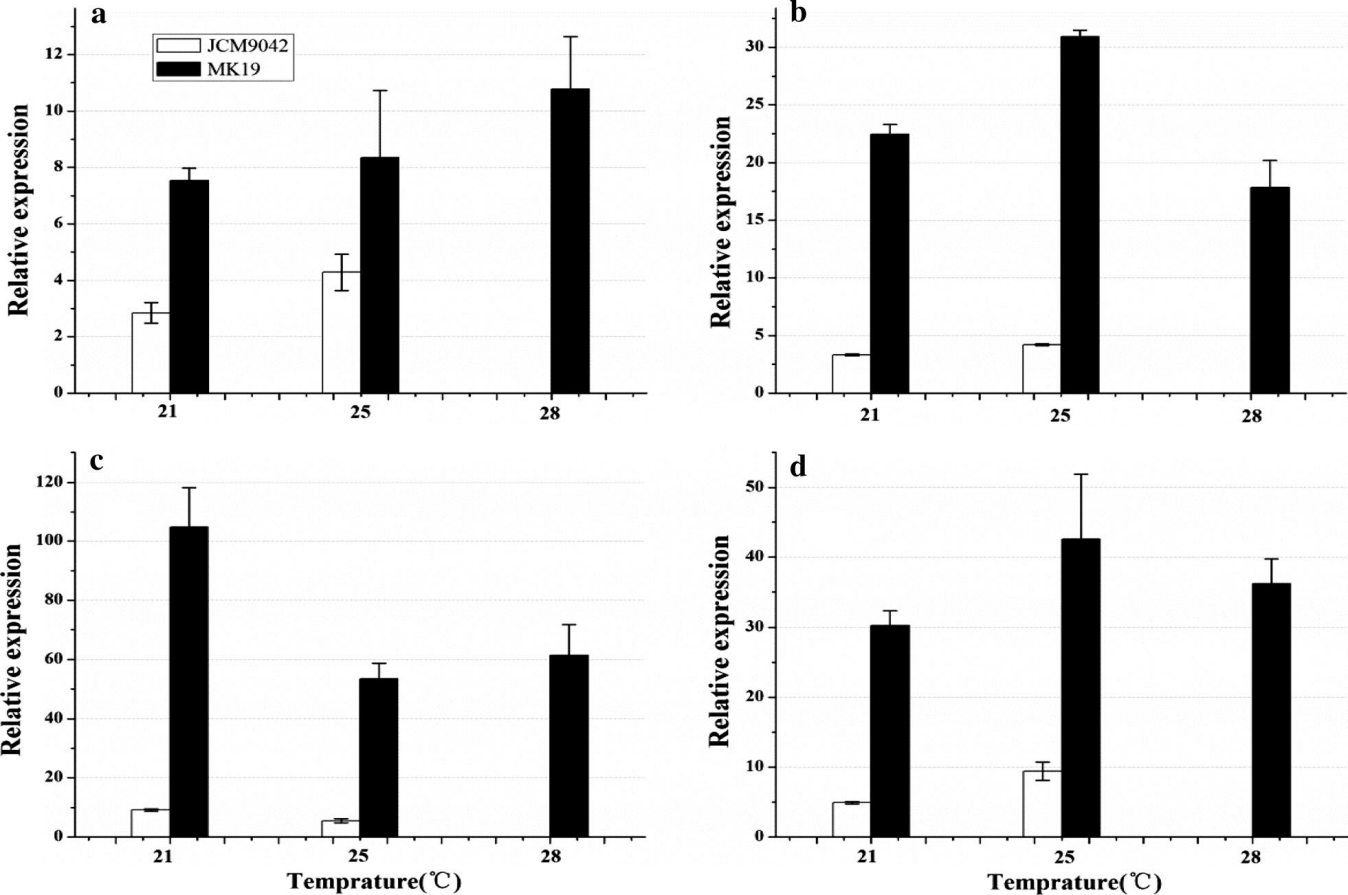
Cellular morphology following culture of JCM9042 grown at 21 °C (**a**), and of MK19 grown at 21 °C (**b**) and 28 °C (**c**)

**Fig. 6:**
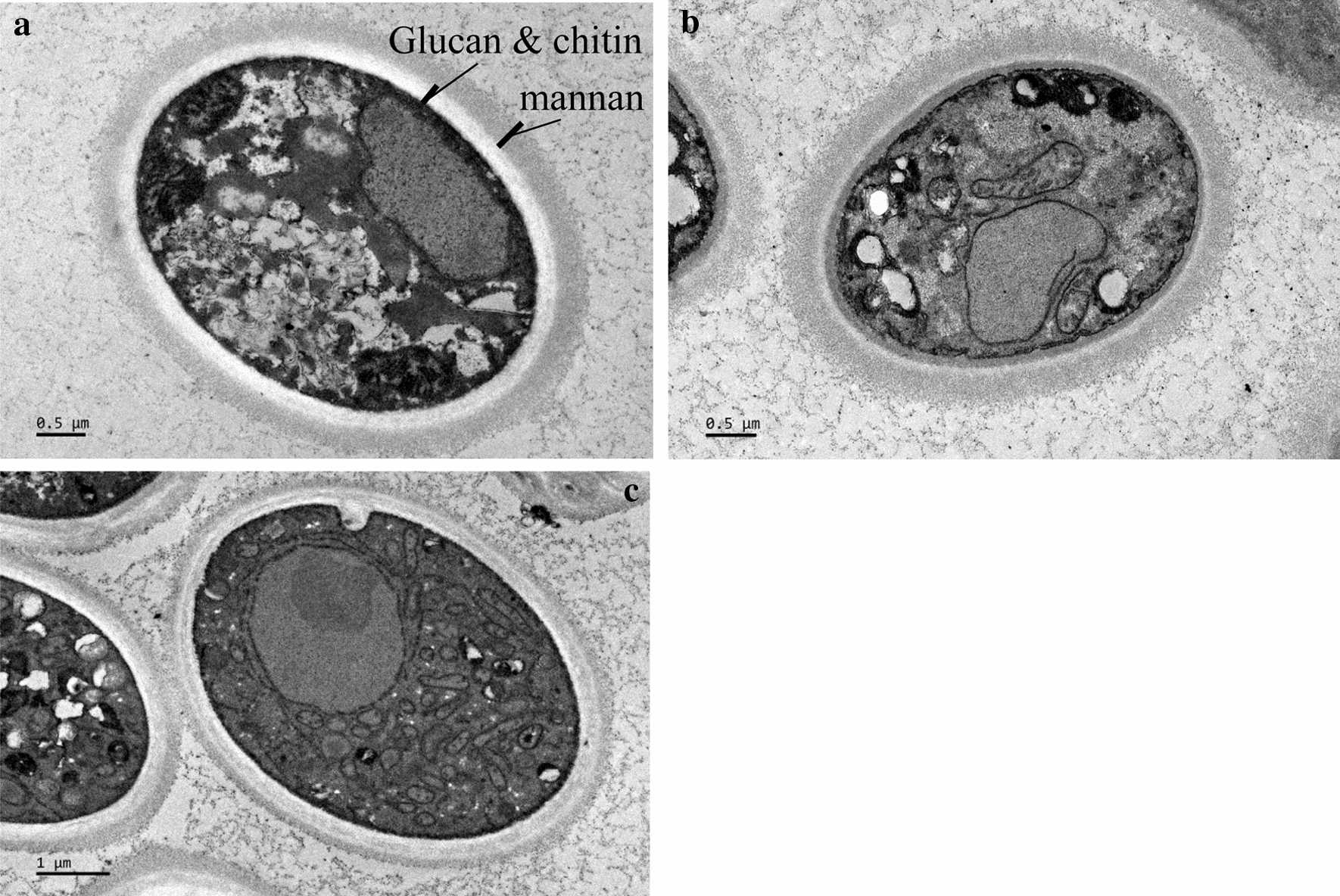
Proportions of three cell wall components of JCM9042 at 21 °C grown and of MK19 grown at 21 and 28 °C

MK19 cell wall structure varied considerably as a function of temperature. Total cell wall thickness was 0.46 ± 0.11 µm at 21 °C and 0.38 ± 0.07 µm at 28 °C. In particular, thickness of the mannan layer at 28 °C (0.15 ± 0.04 µm) was only about half that at 21 °C (0.26 ± 0.08 µm). Thickness of the chitin/ glucan layer increased 0.12 µm at 28 °C (Fig. 5; Table 2). A recent study by H.A. Kang’s group suggests that accumulation of mannan in cell wall enhances stress resistance [31]. In MK19 cell wall outer layer, mannose component was notably reduced at 28 °C (Figs. 5 and 6; Table 2), resulting in disruption of cell wall integrity (CWI), and inhibition of cell growth. In contrast, 28 °C treatment resulted in increased expression of genes associated with mannan component biogenesis; i.e., the genes encoding α-1,3-mannosyltransferase (comp11864_c1), α-1,2-mannosyltransferase (comp12286_c0), and α-1,6-mannosyltransferase (comp11585_c0). MK19 glucan levels were higher at 28 °C, consistent with previous findings that higher β-glucan levels are associated with greater stress resistance in yeast strains [32]. In our study, higher glucan level promoted MK19 survival at 28 °C. In MK19 at 28 °C, mRNAs of most glucan biosynthesis-related genes were downregulated; these included β-1,3-glucanosyltransferase (comp13450_c0), β-1,3-glucanase (comp11848-co, comp11848-c1), and endo-1,3-α-glucanase agn1 (comp12961_c0, comp10598_c0). Yeast cells sometimes deposit more chitin in lateral walls to compensate for compromised cell integrity [32]. In our study, genes that encode enzymes involved in chitin synthesis were upregulated; these include chitin deacetylase (comp13745), chitin synthase CHS1 (comp12056, comp13627), chitin synthase CHS2 (comp12623, comp13980), and chitin synthase CHS2,1,8 (comp14086). Regulatory patterns for glucans and those for mannan were quite different.

**Table 2:** Cell wall thickness comparisons for MK19 grown at 21 and 28 °C, and JCM9042 grown at 21 °C

	Total cell wall (µm)	Chitin/ glucan layer (µm)	Mannan layer (µm)
MK19			
21 °C	0.46 ± 0.11	0.18 ± 0.05	0.26 ± 0.08
28 °C	0.38 ± 0.07	0.24 ± 0.04	0.15 ± 0.04
JCM9042			
21 °C	0.48 ± 0.13	0.16 ± 0.05	0.32 ± 0.08

#### Expression of MVA pathway and astaxanthin pathway genes

The MVA pathway includes the early steps of terpenoid synthesis. *hmgr* and *hmgs*, the key regulatory genes in the terpenoid pathway in eukaryotes, are subject to feedback control at multiple levels; e.g., transcriptional, translational, and enzyme stability [33]. RNA-Seq analysis of MK19 showed no notable expression changes for genes upstream of MVA pathway at 21 vs. 28 °C. The same was true for RT-PCR analysis, except in the case of *idi*, the gene that encodes the enzyme isopentenyl diphosphate (IDP) isomerase. *idi* transcription was induced at 25 and 28 °C in both WT and MK19. For MK19, the maximal increase at 28 °C was ~ 10-fold higher than at 21 °C (data not shown). *hmgs* and *hmgr* expression was not inhibited at 28 °C in MK19, despite the fact that ergosterol content was 2-fold higher at this temperature than at 21 or 25 °C. *hmgs* expression was enhanced at temperatures > 21 °C in both WT and MK19.

**Fig. 7:**
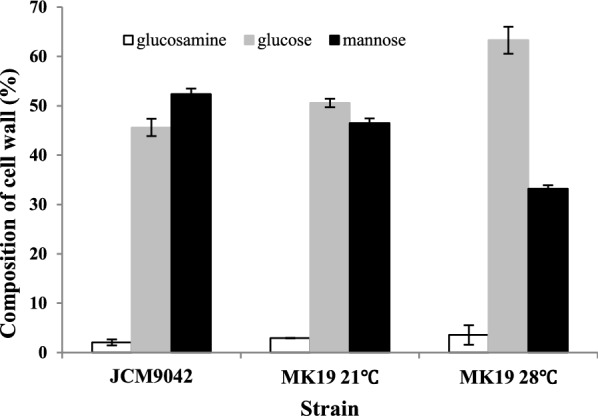
Relative expression of carotenoid pathway genes (**a**: *crt*E; **b**: *pbs*; **c**: *crt*I; **d**: *ast*) in JCM9042 and MK19 grown at three temperatures

Relative expression of carotenoid pathway genes at these three temperatures were compared between WT and MK19. The results (Fig. 7) were consistent with those from RNA-Seq analysis. Expression of *pbs* and *ast* did not differ notably between three temperatures in WT and MK19. *crt*E expression was slightly higher at 28 °C, whereas *crt*I expression was reduced ~ 1.5-fold at temperatures > 21 °C in both WT and MK19.

The inhibitory effect of high temperature on astaxanthin synthesis was exerted mainly at the four dehydrogenation steps leading from phytoene to lycopene in both WT and MK19. Reduced mRNA content of *crt*I was an important cause of astaxanthin inhibition at temperatures > 25 °C. *crt*I mRNA level was > 10-fold higher in MK19 than in WT. This level in MK19 was reduced 1.5-fold at 25 and 28 °C, but was still sufficient to counteract the inhibitory effect of higher temperature and lead to efficient transfer in the four from phytoene-to-lycopene dehydrogenation steps. Therefore, metabolic engineering of these steps, e.g., overexpression of *crt*I, is a feasible and promising method for enhancement of astaxanthin content in WT or other *P. rhodozyma* strains.

## Discussion

The yeast *Phaffia rhodozyma* has great potential for industrial-scale astaxanthin production, and has been a subject of great biotechnological interest for several decades [13, 14]. It is a psychrophilic (low temperature preferring) species whose native habitats in Japan and the North American West Coast are isolated and fairly cold. Applicability of WT *P. rhodozyma* in industrial production is limited because its temperature range for optimal cell growth and astaxanthin biosynthesis is 17–21 °C. We previously established an astaxanthin-overproducing, moderate-temperature mutant strain of *P. rhodozyma*, termed MK19, by NTG and Co^60^ mutagenesis [23, 24]. The regulatory effects of temperature on cell growth and astaxanthin synthesis of *P. rhodozyma* have not been investigated, and our knowledge of the molecular regulatory mechanisms in general is quite fragmentary.

We describe here for the first time the biosynthetic and regulatory mechanisms of cell growth and astaxanthin production at high temperature (28 °C) in *P. rhodozyma*. WT strain cannot grow at 28 °C. MK19 biomass production at 28 °C reached 6 g/L, which was 80% less than values at 21 or 25 °C. Our RNA-Seq and metabolic analyses revealed 5 regulatory patterns involving temperature, as follows. (1) Upregulation of genes that encode proteins involved in protein folding and stabilization, including HSPs, rRNA processing, and amino acid synthesis. These genes have high copy numbers, reflecting protein denaturation and misfolding, which may result from 28 °C stress. The high copy numbers help mitigate such stress through increased protein synthesis, and refolding and reactivating functions of denatured proteins. (2) DNA metabolism was strongly suppressed at the mRNA level, and low mRNA content accounted for the low viability of MK19 at 28 °C. (3) Inability of WT to grow at 28 °C was due in part to reduced synthesis of f.a. (a major component) in cell membrane, reflected in low transcriptional capacity of key f.a. synthesis genes (*acc*, *fas*1, *fas*2) and low f.a. content (particularly of 18:1 species). Low biomass of WT at 28 °C was related to altered content of 18:1 species. (4) High content (7500 µg/g) of ergosterol, a structural enhancement (strengthening) component of cell membrane, compensated in part for the low f.a. content, and explain the relief the growth repression of MK19 at 28 °C. Engineering of the f.a. biosynthetic pathway could potentially convert MK19 or other *P. rhodozyma* strains to mesophilic strains having optimal growth temperature ~ 28 °C. (5) Fungi have a rigid cell wall that plays an important role in cell growth. Cell wall in *P. rhodozyma* loses integrity at 28 °C because the outer mannose layer becomes thinner, another reason why WT is unable to grow at this temperature.

**Fig. 8:**
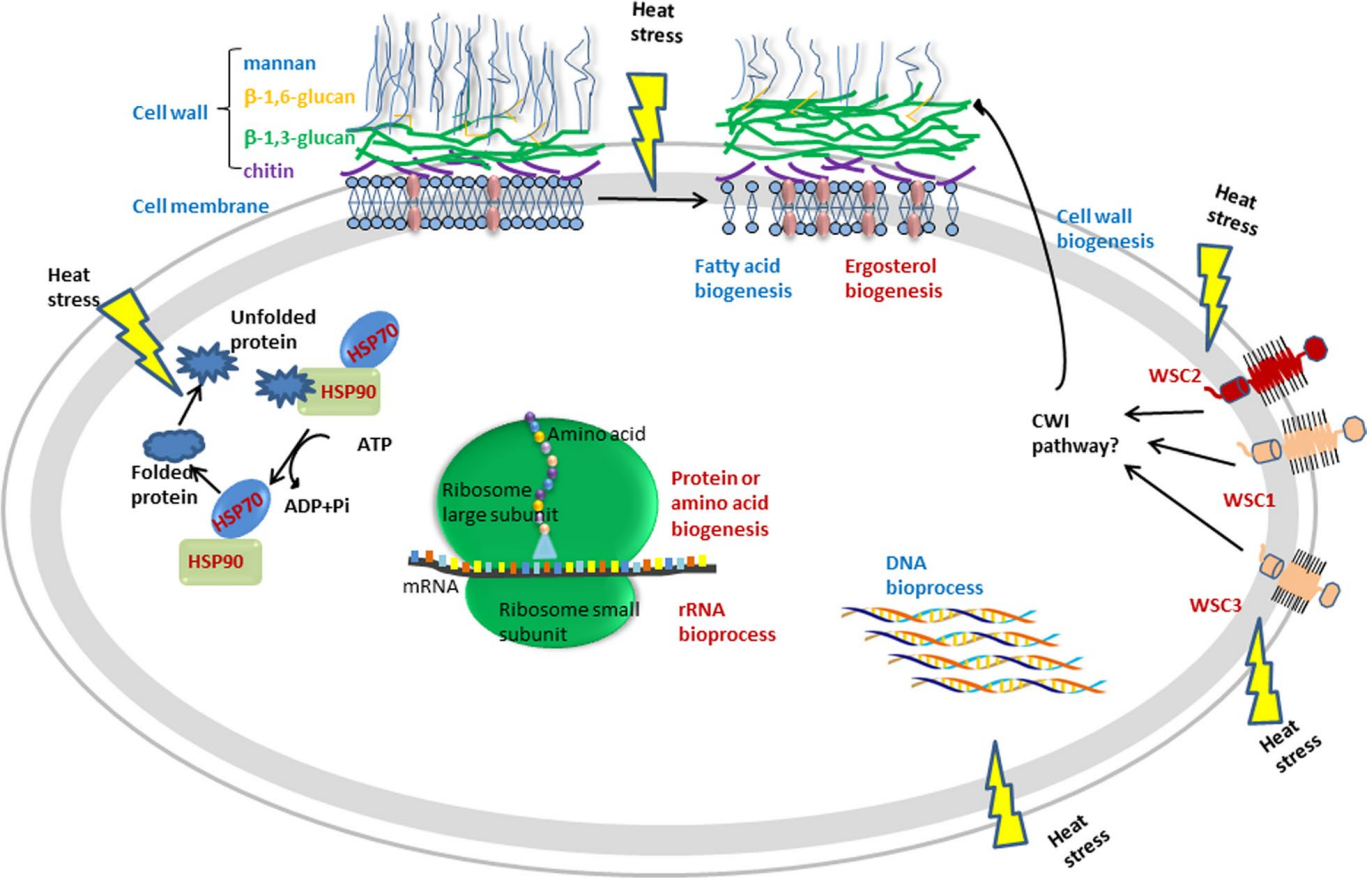
Schematic model of candidate processes contributing to adaptive response of MK19 to 28 °C stress, based on RNA-Seq and metabolic analyses. 28 °C heat stress triggers activation of CWI, HSP pathways, etc. resulting in global transcriptomic changes in various biosynthetic pathways, including cell wall remodeling enzymes, protein folding and stabilization, ergosterol, DNA, fatty acids metabolism

Cell wall plasticity and composition depend on active regulation of underlying biosynthetic and restructuring processes. The cell wall integrity (CWI) pathway is a central signaling cascade that is highly conserved in fungi [30, 31]. CWI pathway is essential for adaptation to a wide variety of cell wall disrupting conditions, including heat stress. Heat shock triggers activation of CWI and HSP pathways, resulting in global transcriptomic changes in various biosynthetic pathways, including cell wall remodeling enzymes and mannan and glucan biosynthesis. Observed differential expression of genes encoding WSC proteins, f.a., and other cell wall components reflects the essential role of CWI and HSP pathways in adaptation to 28 °C stress in MK19. A schematic model of candidate processes contributing to adaptive response of MK19 to 28 °C stress, based on RNA-Seq and metabolic analyses, is presented in Fig. 8.

In summary, regulatory patterns (1) and (4) contributed to tolerance of cell growth, while (2), (3), and (5) accounted for damage of cell growth of MK19 at 28 °C.

In our 2011 study, astaxanthin content of WT *P. rhodozyma* was lower at 25 °C than at 21 °C whereas ergosterol content was the same at these two temperatures, indicating that astaxanthin and ergosterol are regulated through independent mechanisms [24]. Synthesis of carotenoids (but not of other isoprenoids) was inhibited at 25 °C. *crt*I mRNA level at 25 °C, which was ~ 1.5-fold lower than that at 21 °C, accounted for such inhibition in WT.

Despite the transcriptional inhibition of *crt*I, the high *crt*I mRNA of MK19 (> 10-fold higher than that of WT) was enough and accounted for the high efficiency of astaxanthin synthesis at 25 or 28 °C. Thus, inhibition of astaxanthin synthesis by high temperature in *P. rhodozyma* appears to occur mainly at the conversion step from phytoene to lycopene. Metabolic engineering of this step (e.g., overexpression of *crt*I) could be a feasible method for increasing astaxanthin content at temperatures > 25 °C in this species.

Astaxanthin and ergosterol content in MK19 were increased > twofold at 28 °C, in contrast to cell growth, suggesting that isoprenoid biosynthetic pathway (particularly MVA pathway which includes the initial steps of isoprenoid synthesis) was activated at this temperature. There is competition among intermediate compounds of the f.a., astaxanthin, and ergosterol pathways because these macromolecules share common precursors, such as acetyl-CoA, etc. Astaxanthin content is inversely correlated with f.a. synthesis in *P. rhodozyma* [23]. F.a. synthesis in MK19 was inhibited at 28 °C, resulting in a probable accumulation of acetyl-CoA and other precursors, enhanced activity of MVA pathway, excessive synthesis of ergosterol, and reduction of biomass. Precursors from MVA pathway and F. a. synthesis were also diverted to astaxanthin biosynthesis via activated carotenoid pathway in MK19, resulting in increase of astaxanthin content to 2000 µg/g. The present findings, taken together, clearly indicate that strengthening of the MVA pathway is a feasible and efficient metabolic engineering approach for enhancement of astaxanthin synthesis in MK19 or other moderate-temperature strains.

## Conclusions

The inability of growing at 28 °C in WT *P. rhodozyma* is due to blocking of DNA, RNA, cell membrane, and cell wall biosynthesis through transcriptional regulation. In the moderate-temperature mutant strain MK19, excessive accumulation of ergosterol, glucan, and protein, at this temperature led to biomass production of 6 g/L. Precursors, such as acetyl-coA, from impeded f.a. pathway, were diverted to and stimulated both of astaxanthin and ergosterol biosynthesis. Strengthening of the MVA pathway could be a feasible metabolic engineering approach for enhancement of astaxanthin synthesis in MK19 or other moderate-temperature producer strains.

## Methods

### Strains and culture conditions

WT *P. rhodozyma* strain JCM9042 was from the Institute of Physical and Chemical Research, Japan. MK19, an astaxanthin-overproducing and moderate-temperature mutant strain, was generated and screened by NTG and Co^60^ mutagenesis in our laboratory [24]. Both strains were maintained on potato dextrose agar slants at 4 °C.

Seed medium and fermentation medium were prepared as described previously [24]. All experiments were conducted in shaking flask culture in 250 ml flasks containing fixed liquid volume 25 ml.

WT and MK19 cells were transferred from 4 °C slants to fresh slants and maintained at 21–24 °C for 72 h. Loopfuls of lawn were inoculated in seed medium and incubated at 21–24 °C for another 72 h. Five percent of the preincubation broth was further inoculated for another 36 h to produce starter culture. The overall production period of fermentation culture was 4–6 days on a rotary shaker (210 rpm, 21 °C), with samples taken at 12 or 24 h intervals. All experiments were performed in triplicate or quadruplicate.

Cell concentrations were estimated by measuring optical density at wavelength 600 nm. Dry weight of cells was determined by centrifuging 35 ml broth at 12,000 rpm, rinsing with distilled water, and drying at 85 °C until attaining constant weight (~ 15 h).

### Astaxanthin and total ergosterol measurement

One ml broth was centrifuged at 12,000 rpm for 1 min and washed with distilled water. Pellets were mixed with 200 µl dimethyl sulfoxide preheated to 70 °C, stirred, and the mixture was heated in a water bath for 20 min at 70 °C. Broken cells were extracted with methanol/ dichloromethane (3:1), agitated, and centrifuged at 2000 rpm. The supernatant was transferred to another tube. This process was repeated until pellets showed no red color. Astaxanthin and free ergosterol were analyzed quantitatively by HPLC on a C18 column (250 × 4.6 mm; 5 µm; Chuangxintongheng, Beijing): temperature 40 °C, flow rate 1.0 ml/min, wavelength 476 nm (for astaxanthin) or 280 nm (for ergosterol). The mobile phase consisted of methanol 97%/ water 3%. Astaxanthin and ergosterol were identified based on retention time in comparison with standard astaxanthin (Sigma-Aldrich; Shanghai).

### DNA, RNA, protein, fatty acids measurement

The cell samples were collected by centrifugation (12,000 rpm, 2 min) and frozen in liquid nitrogen immediately. then cell lysis were obtained by shattering thoroughly by homogenizer for 5 min, protein samples were collected by centrifugal (12,000 rpm, 20 min) after adding 20 mM PBS buffer (pH 7.0) to cell lysis, protein content of supernatant were determined by Bradford method. The DNA and RNA samples were extracted by HiBind DNA Mini Column and HiBind RNA Mini Column from DNA or RNA extraction kit (Omega, US), after several washing steps, the DNA or RNA was eluted from the column. The DNA, RNA, Protein content were determined by Nanodrop 2000 (Thermo), the fatty acid content was determined the same as Miao et al. [23].

### Total RNA purification and reverse transcription

Cells of WT and MK19 in 1 ml broth were harvested by centrifugation (12,000 rpm, 1 min), frozen immediately in liquid nitrogen, and stored at − 70 ºC until processing. RNA extraction was performed using TRIzol Reagent (Invitrogen) as per manufacturer’s instructions. Total RNA concentration was determined by spectrophotometry at 260 nm. Aliquots of extracts were subjected to agarose gel electrophoresis to check RNA integrity.

To degrade trace amounts of genomic DNA in RNA preparations, DNase I (Takara Japan) (1 µl; 5 U/µl) was added to reaction mixture containing 5 µg RNA, 4.4 µl of 25 mM MgCl_2_, 2 µl of 10 × buffer (Takara Japan), and DEPC-treated water was added to give total volume 20 µl. Reaction mixture was incubated for 30 min at 37 °C followed by heat denaturation for 15 min at 65 °C.

RNA sample was heated to 70 °C, and reverse transcription reaction was performed in a final volume of 25 µl containing 2 µg total RNA, 1 µl oligo(dT) primer, 0.5 mM dNTPs, and 200 U M-MLV reverse transcriptase H minus (Promega). Reaction mixture was incubated for 60 min at 42 °C, then heated to 65 °C for 15 min.

### Real-time polymerase chain reaction (RT-PCR)

RT-PCR analyses were performed with an ABI 7900HT apparatus (Applied Biosystems; Norwalk, CT, USA), using RNA samples as template. Dissociation curves were constructed to test amplification validity. Target genes were obtained from NCBI (ncbi.nlm.nih.gov/). Database accession numbers and corresponding primer sets in RT-PCR are as listed in Miao et al. [24]. *actin* was used as control gene. Relative gene expression was calculated by 2^−***△△****CT*^ (cycle threshold) method using Sequence Detection software program v1.2.2 (Applied Biosystems). Each RT-PCR analysis was run in triplicate or quadruplicate to test consistency.

Transcriptome analysis.

JCM9042 and MK19 were grown in synthetic medium for 48 h at 21 or 28 °C. Subsequent procedures for RNA sequencing were conducted by Majorbio Bio-Pharm Technology (Shanghai). Clean data were de novo assembled by Trinity software program (github.com/trinityrnaseq/trinityrnaseq/wiki). Genes with fold changes > 1.5-fold were functionally classified using the Munich Information Center for Protein Sequences (MIPS) FunCat. Processed data were deposited in NCBI Sequence Read Archive (SRA) under accession number SUB7646115.

### Transmission electron microscopy (TEM) of cell surface

Samples were cultured and collected under the same conditions as for RNA-Seq samples, fixed in 2% glutaraldehyde for 2 h, then subjected for 1.5 h to second fixation with 1.5% potassium permanganate solution, rinsed twice (7 min each time) with PIPES 0.1 M, washed by ethanol gradient (30, 50, 70, 85, 95%) elution, washed three times (10 min each time) with 100% ethanol, soaked in acetone/ Spurr resin (ratios 3:1, 1:1,1:3; 2 h each time), and embedded overnight in Spurr resin. Images were recorded by TEM using a Tecnai G2 Spirit Twin microscope (FEI) at acceleration voltage 120 kV.

### Statistical analysis

Student’s *t*-test was performed using SPSS (Statistical Program for Social Sciences) software program.

## Data Availability

The transcriptional data was deposited to the NCBI Sequence Read Archive (SRA), other datasets and strains generated from present study are available from the corresponding author on a reasonable request.

## Abbreviations

hmgr: 3-hydroxy-3-methylglutaryl-CoA reductase
hmgs: 3-hydroxy-3-methylglutaryl-CoA synthase
crtE: GGPP synthase
crtI: Phytoene dehydrogenase
pbs: Phytoene-β-carotene synthase
ast: Astaxanthin synthase
idi: IDP isomerase
mvk: Mevalonate kinase
mpd: Mevalonate pyrophosphate decarboxylase
fps: FPP synthase
acc: Acetyl-CoA carboxylase
sqs: Squalene synthase
